# Genome-wide association studies of *C. trachomatis* identify novel tissue tropism-predicting loci and sequences that discriminate among major genome sequence clades

**DOI:** 10.1128/iai.00266-26

**Published:** 2026-07-17

**Authors:** Robert J. Suchland, Stephen A. Ramsey, Valerie Tran, Steven J. Carrell, Xisheng Wang, Kevin Hybiske, Daniel D. Rockey

**Affiliations:** 1 Division of Allergy and Infectious Diseases, Department of Medicine, University of Washington 7284, Seattle, Washington, USA; 2 Department of Biomedical Sciences, Oregon State University, Corvallis, Oregon, USA; 3 Center for Quantitative Life Sciences, Oregon State University, Corvallis, Oregon, USA; University of California Davis, Davis, California, USA

**Keywords:** *Chlamydia*, tropism, genomics, sexually transmitted, trachoma

## Abstract

*Chlamydia trachomatis* infections cause chronic and debilitating diseases of the eye and genital tract. Recent data suggest that infections are common at other tissue sites, including the lower intestinal tract of both men and women. Our previous work demonstrated tropism-predicting sequences in male rectal isolates, with modification in the PmpE sequence and predicted structure being highly correlated with male rectal infection. An expanded collection of strains from five different tissue sites was used to identify novel tropism-predicting markers for the different tissues. These data demonstrate that *C. trachomatis* strains colonizing the male rectum are unique with respect to all other tested sites. Additionally, ocular strains have a consistent set of genes that separate them from strains isolated from any other tissue. Novel polymorphisms predictive of ocular tropism include a single indel in *mrcA* and a region between *pmpH* and *pmpI*, including the hypothetical gene *ct873*. These analyses also identified a novel urogenital clade, termed the variable-OmpA clade, in which strains carry a variety of *ompA* sequences in an otherwise shared genomic background. This clade is defined primarily by single-nucleotide polymorphisms (SNPs) within and adjacent to the highly variable plasticity zone. Collectively, this work explores the continuing evolution of *C. trachomatis* strains and clades in a genomic background that only engages in intraspecies horizontal gene transfer.

## INTRODUCTION

The genus *Chlamydia* includes a variety of different pathogens of humans and other animals. Many aspects of biology and pathogenesis are similar between different *Chlamydia* spp., including an obligate intracellular developmental cycle, alternating developmental forms in a productive infection, similar virulence strategies, and a reduced genome (1, 2). Genome sequence analysis has identified four deep lineages within the species comprising the Lymphogranuloma Venereum (LGV) biovar, the trachoma biovar, and two urogenital biovars termed Prevalent (T1) and Non-Prevalent (T2) clades (3–5). *Chlamydia trachomatis* is a serious human pathogen that causes disease at two main tissue sites: the conjunctivae and the female genital tract (6). Gastrointestinal infections by urogenital strains of the pathogen are of clinical importance, as such infections may serve to facilitate persistence in both men and women (7). The LGV biovar contains strains that are associated with aggressive pathologies of the rectum and lymph nodes and is most commonly observed in men who have sex with men (8).

Horizontal gene transfer (HGT) is common at homologous loci in clinical *C. trachomatis* strains, but evidence of recent genetic exchange from organisms outside of the genus is absent (3, 9). Therefore, virtually all contemporary strategies for evolution in this genus are a function of mutation and intraspecies HGT. Whole genome sequencing and phylogenetic tree analysis of hundreds of clinical strains, from both the University of Washington *Chlamydia* Repository and the published genome databases, have led our group (10) and others (11) to propose distinct models of *ompA-* and *pmpE-*focused HGT in strains isolated from ocular, cervical, and rectal anatomic sites of infection.

We previously demonstrated that *C. trachomatis* isolates collected from the male rectum had a unique genomic fingerprint relative to those isolated from the female genital tract (10). In this work, we expand our collection of genomes to include isolates cultured from each of five different human tissue sources: the cervix, male and female rectum, the male urethra, and the eye. The data demonstrate that the previously identified male rectal genotype of *C. trachomatis* is restricted to isolates that infect that tissue, with female rectal and male urethral strains sharing more similarity to strains isolated from the cervix. These studies also define a novel phylogenetic branch termed the variable-OmpA clade (VO clade), defined by the presence of a distinct non-prevalent urogenital backbone with a variety of *ompA* acquired by HGT from ocular, urogenital, or rectal donor strains. The VO clade is represented by geographically and temporally distinct clinical *C. trachomatis* cultures and patient specimens. Many of the isolates in this clade have shared D/UW-3-like sequence from the *Chlamydia* plasticity zone (PZ: *ct154-ct174*), a region of the chromosome that is highly variable among strains. Collectively, these models offer mechanisms by which *C. trachomatis* exploits recombination and selection to evolve for optimal growth and/or dissemination to different tissue sites.

## RESULTS

### Genome-wide association study (GWAS) to identify tissue-tropic loci

**Fig 1:**
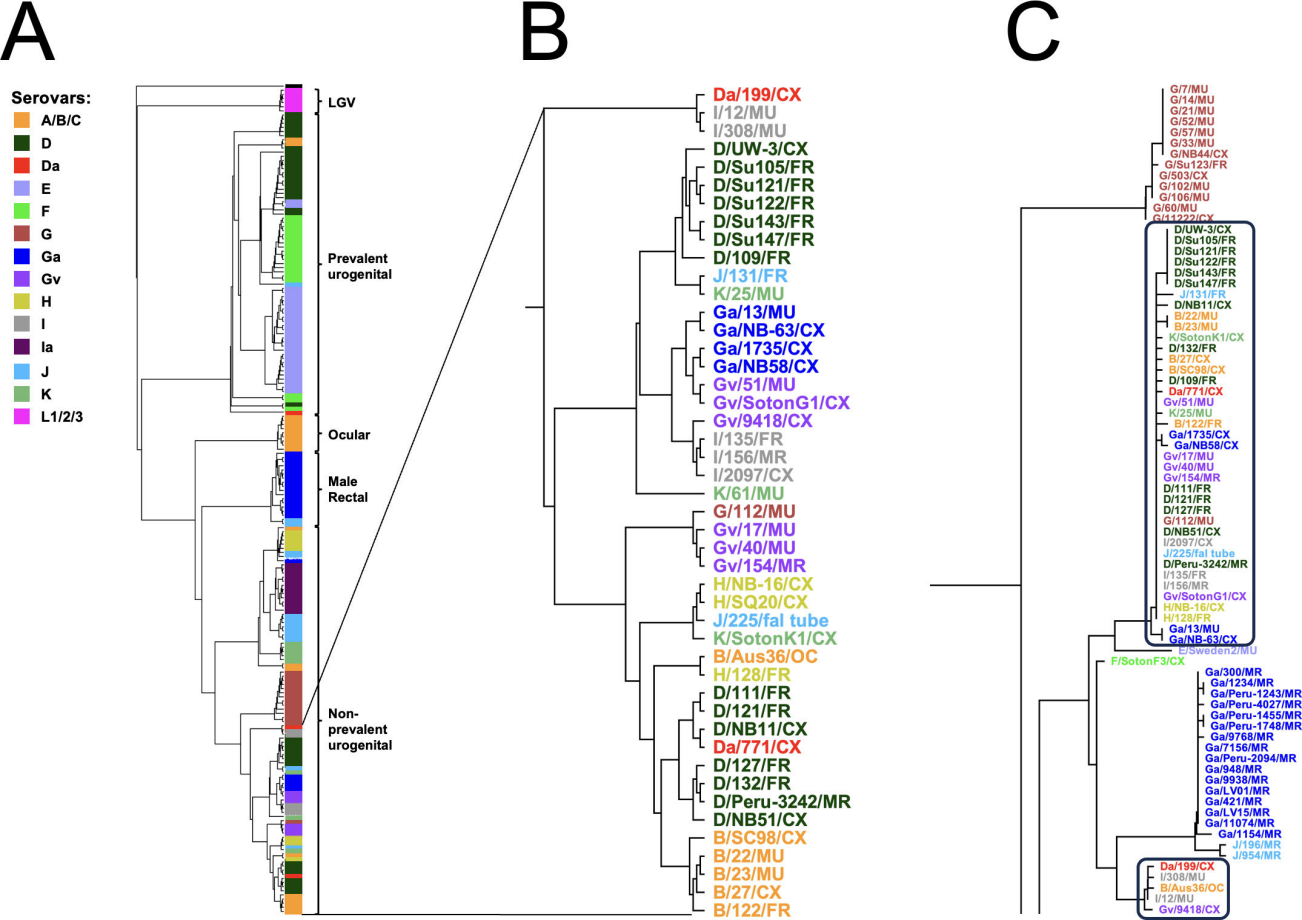
Neighbor-joining-based phylogenetic tree of 200 *C. trachomatis* chromosomal sequences. Panel **A**: The full tree, with strains color-coded by serovar/genovar. A full tree with strain names included is found in Fig. S1, and all strains are described in Table S1. Panel **B**: Strains within the chromosomal tree that fall into the VO clade. Panel **C**: A section of a tree of the *C. trachomatis* PZ, with strains in the VO clade identified with boxes. A complete PZ tree is shown in Fig. S4. Color coding for all panels is based on the legend in Panel A.

Our previous work associated a limited set of coding sequences with tropism to the male rectum (10). For the current study, we included 108 additional genome sequences from strains collected at five tissue sites to determine if the tropism loci identified previously were correlated with both female and male rectal tropism. A total of 200 genome sequences were analyzed, including strains from the female reproductive tract (*N* = 68), the female rectum (*N* = 40), the male rectum (*N* = 37), the male urethra (*N* = 35), lymph node (LGV: 6), and the ocular (*N* = 14) sites of infection (Table S1). Novel strains were genome-sequenced, and a full genome phylogenetic tree was built using a neighbor-joining-based strategy (Fig. 1A; Fig. S1). The overall characteristics of the tree were consistent with our work and that of others (3, 5, 10).

**Fig 2:**
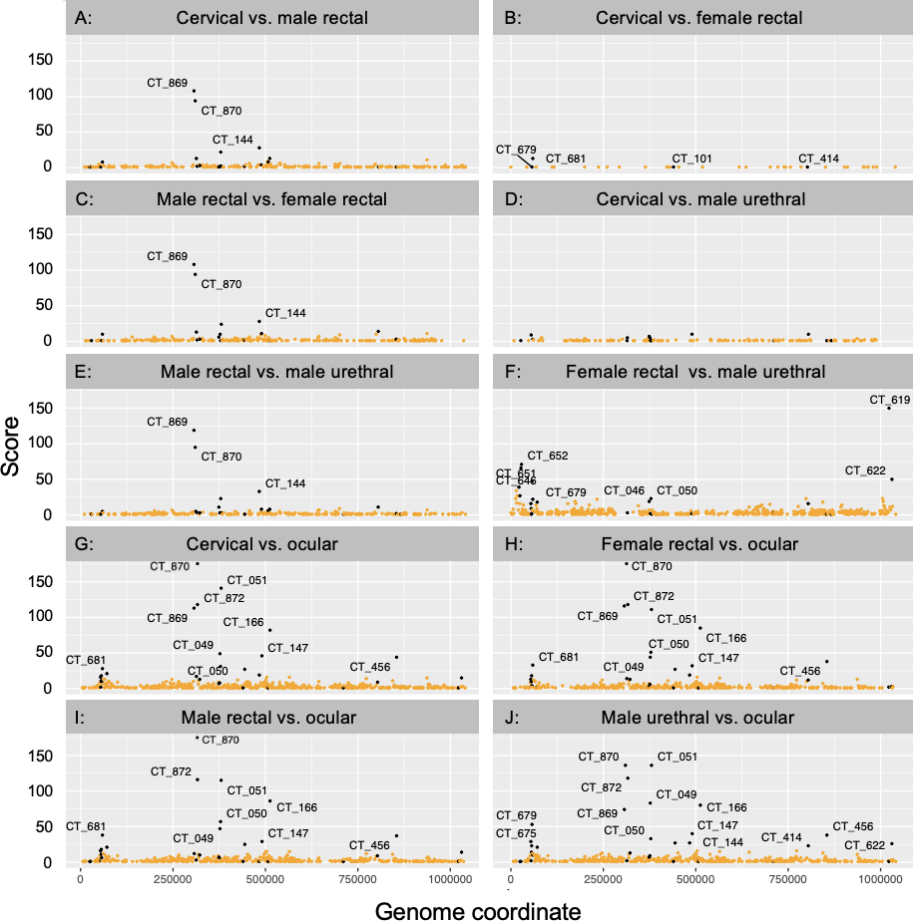
(**A–J**) GWAS-based single-nucleotide polymorphism (SNP) counts for *C. trachomatis* open reading frames (ORFs) in strains collected from cervical, ocular, male urethral, male rectal, and female rectal sites of infection. Data points represent the number of significant SNPs per gene, corrected for the length of the gene in kb. The horizontal axis is position in the chromosome, starting at *hemB* (*ct633*), with the scale indicated in base pairs. The vertical axis shows the significant SNPs per ORF, divided by the length of the ORF in kbp.

A GWAS-based approach was then used to evaluate correlations between allelic differences and strains sourced from each tissue site. The data were first evaluated for their consistency with our prior analysis of male rectal vs. cervical strains, and the current data supported our previous results. The genes *pmpE* (*ct869*), *pmpF* (*ct870*), and the hypothetical gene *ct144* remained strong predictors of male rectal site of infection, while the correlation between the *ct050* sequence and tissue site was reduced in significance (Fig. 2A; Table 1; Table S2).

**TABLE 1:** The most significant hits for each tissue comparison examined in this study

CDS	ORF/Gene	Tissue comparison	Most significant *P*-values	SNPs per gene
CT_049	*pls1*	Cervical – Ocular	3.08E-09	83
		Male Urethral – Ocular	4.26E-07	83
CT_050	*pls2*	Male Rectal – Ocular	2.85E-07	57
		Female Rectal – Ocular	1.61E-07	71
CT_051	hypothetical protein	Cervical – Ocular	4.25E-07	141
		Male Rectal – Ocular	2.85E-07	115
		Male Urethral – Ocular	4.26E-07	136
		Female Rectal – Ocular	3.43E-06	111
CT_144	hypothetical protein	Cervical – Male Rectal	4.52E-11	28
		Female Rectal – Male Rectal	6.269E-08	28
		Male Urethral – Male Rectal	6.263E-10	33
CT_166	Tox/adhesin	Cervical – Ocular	3.08E-09	82
		Male Rectal – Ocular	2.85E-07	86
		Male Urethral – Ocular	4.26E-07	80
		Female Rectal – Ocular	1.613E-07	85
CT_619	Type III protein	Male Urethral – Female Rectal	0.007	150
CT_651	Exp. lipoprotein	Male Urethral – Female Rectal	0.007	66
CT_652	*recD2*	Male Urethral – Female Rectal	0.007	71
CT_679	*tsf*	Male Urethral – Ocular	1.8684E-05	53
CT_869	*pmpE*	Cervical – Male Rectal	6.38E-13	108
		Female Rectal – Male Rectal	1.596E-11	108
		Male Urethral – Male Rectal	1.334E-10	119
		Cervical – Ocular	8.7E-11	113
		Male Urethral – Ocular	3.4521E-09	74
		Female Rectal – Ocular	1.023E-09	116
CT_870	*pmpF*	Cervical – Male Rectal	6.38E-13	94
		Female Rectal – Male Rectal	4.229E-10	94
		Male Urethral – Male Rectal	1.334E-10	95
		Cervical – Ocular	8.7E-11	186
		Male Rectal – Ocular	2.09E-09	179
		Male Urethral – Ocular	3.4521E-09	136
		Female Rectal – Ocular	1.023E-09	185
CT_872	*pmpH*	Cervical – Ocular	5.25E-11	118
		Male Urethral – Ocular	3.4521E-09	118
		Female Rectal – Ocular	1.023E-09	118

GWAS was then used to demonstrate that female rectal strains are much more similar to isolates collected from the cervix, while the same three genes—pmpE (*ct869*), *pmpF* (*ct870*), and the uncharacterized *ct144*—remained predictive for comparing male rectal strains from either cervical or female rectal strains (Fig. 2A through C). A comparison of strains isolated from the male urethra with each other’s tissue source was also conducted. These data demonstrated that male urethral strains were distinct from those isolated from the male rectum and were much more similar to strains isolated from the female rectum and cervix (Fig. 2D through F). Each of *pmpE*, *pmpF*, and *ct144* remained highly predictive of male rectal vs. male urethral strains. The comparison of ocular strains with each other tissue led to a very similar set of tropism-predicting sequences (Fig. 2G through J), with the one exception that *pmpE* does not discriminate between ocular and male rectal strains (Fig. 2I).

### Multivariate analysis of the GWAS results

**Fig 3:**
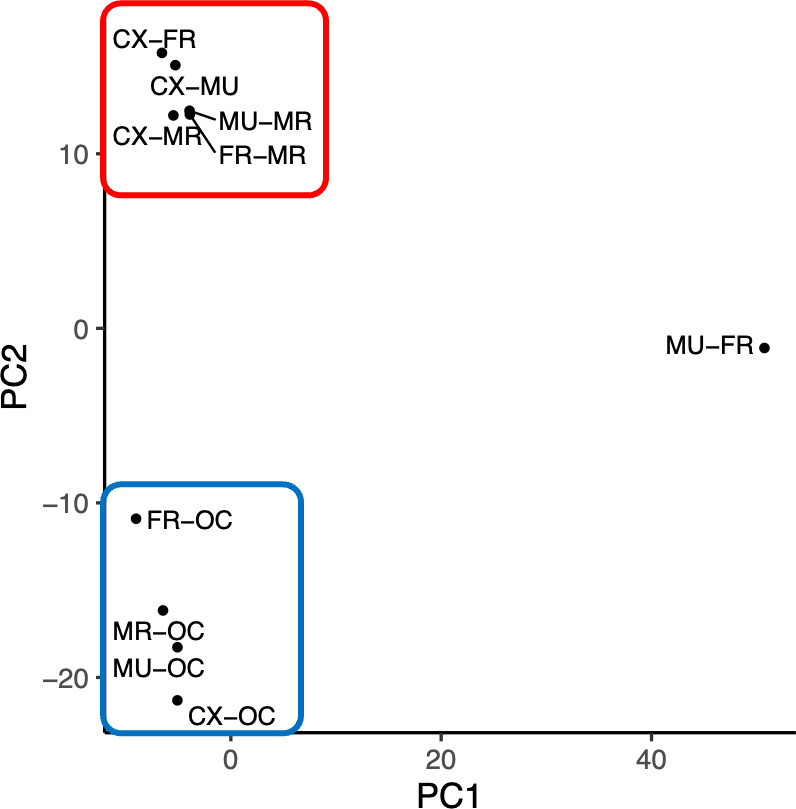
Principal component analysis scores plot of tissue comparisons in Fig. 2. Tissue site abbreviations are as follows: CX, female cervical; FR, female rectal; MR, male rectal; MU, male urethral; OC, ocular. Each of the 10 data points corresponds to a tissue type comparison (e.g., MR-OC corresponds to a comparison between *C. trachomatis* samples obtained from infections of the male rectum and ocular tissue). Each tissue type comparison corresponds to one of the GWAS analyses for which gene-level significance data were shown in Fig. 2. The red and blue rounded-corner boxes indicate sets of five and four (respectively) tissue type comparison GWAS analyses that were tightly grouped.

A quantitative evaluation of the gene-level differential significance profiles across the 10 tissue comparisons was conducted using a principal component analysis (PCA)-based approach. The resulting PCA scores of the first two principal components identified a clear difference between the clustering of the ocular vs. nonocular comparison profiles and each other profile (Fig. 3). The cluster involving ocular strains is well-separated from all comparisons involving cervical or male rectal by principal component 2, with the difference being statistically significant in a multivariate difference-of-means test (*P* < 2.76× 10^-6^ , permutation-tested Hotelling’s $T^{2}$). The major outlier in these comparisons is female rectal vs. male urethral, which is distinct from each other in comparison, both in the GWAS plot (Fig. 2F) and the PCA (Fig. 3).

### Single indels or short sequences that predict ocular tropism

**Fig 4:**
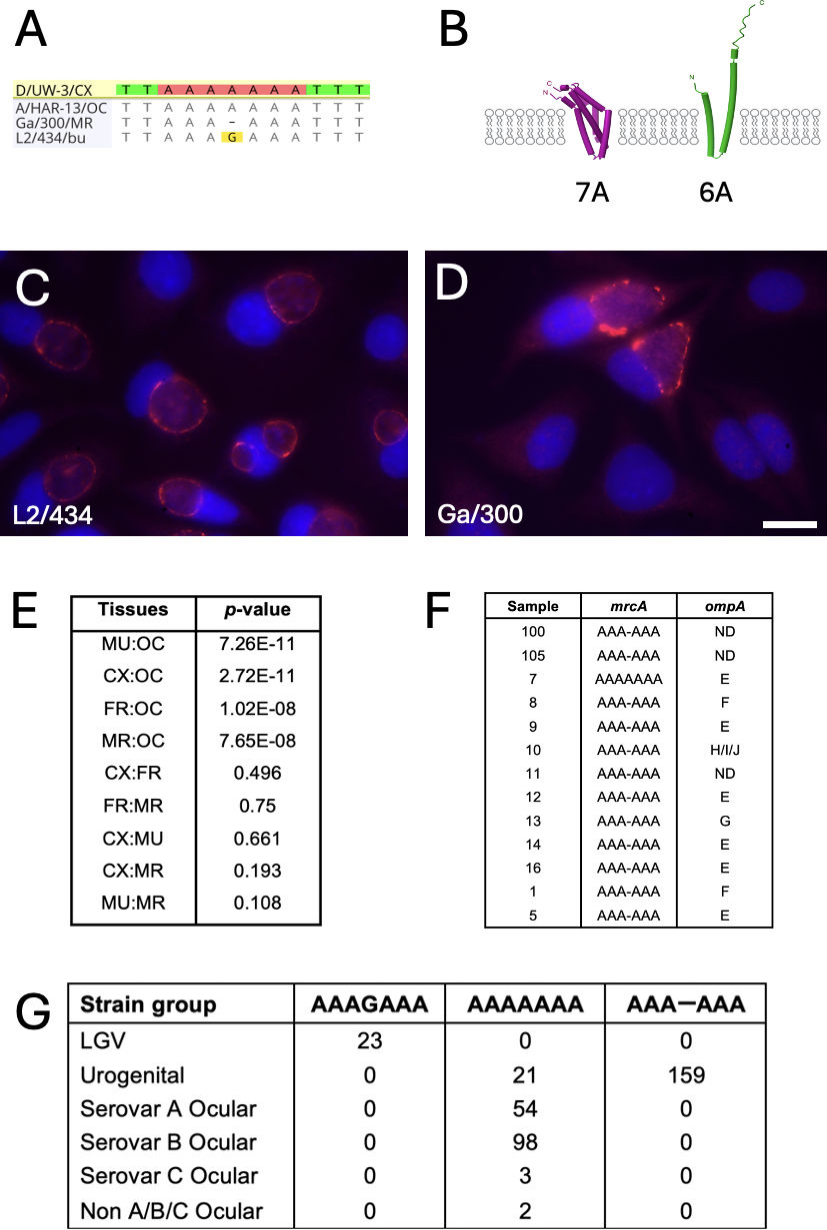
A single indel in *mrcA* (*ct101*) is predictive of tropism to the ocular site of infection. Panel **A**: Variation in sequence at the poly-A repeat (positions 235–242) within three strains of *C. trachomatis*. A full alignment for this sequence is found in Fig. S2. Panel **B**: Alphafold3 modeling of the predicted sequences from A/HAR-13 (7A) and Ga/300 (6A). Strains within the LGV cluster have an internal G within the poly-A sequence, but the overall reading frame is intact, and the protein has very similar sequence and structural predictions. Panels **C and D**: Localization of MrcA (red) in McCoy cells infected with LGV-434 (AAAGAAA strain, panel **C** and Ga/300 (AAAAAA strain, panel **D**. Nuclear DNA is labeled with 4′,6-diamidino-2-phenylindole (DAPI, blue). The scale bar in F indicates 10 microns for each panel. Panel **E**: Predictive value of the *mrcA* indel for each tissue comparison in the GWAS. Panel **F**: PCR-generated sequence surrounding the indel collected from uncultured clinical specimens. Parallel sequencing of *ompA* was included in these experiments, and the genovars identified are also included for each specimen. Full *mrcA* sequences for each of these specimens are shown in Fig. S3. Panel **G**: Summary of data surrounding the A-repeat sequence in *ct101* for LGV, ocular, and urogenital/rectal strains. All accession numbers for these data are provided in Table S1 or from a study by Hadfield et al. (4).

Each of the GWAS analyses was further assessed by considering the number of significant SNPs per gene, which was then factored to reflect the length in kb of each gene. A second gene-independent approach was used, in which we evaluated significant GWAS predictions associated with single SNPs across the genome, since the gene-centered analysis devalues individual SNPs. Two new tropism-predicting loci were identified using this latter approach. The first of these is an indel within *ct101* (*mrcA*) that is highly predictive for ocular infection (Fig. 4A and E; Fig. S2; Table S2, highlighted in yellow). MrcA is a small inclusion-membrane-localized protein (Inc protein) that is associated with a nonlytic form of chlamydial release from cells, termed extrusion (12–14). The predictive indel is found in an intragenic homopolymeric sequence in which the full-length gene has seven adenosines, while the alternate allele has six (Fig. 4A; Fig. S2). This alteration leads to a shorter predicted protein that is very nearly identical upstream of the indel but is truncated and fully dissimilar after the indel (Fig. 4B, Fig. S2). Antisera developed against a peptide in the amino terminal half of MrcA (12) demonstrate that strains with full-length or truncated are both localized to the inclusion membrane within infected cells (Fig. 4C and D).

Our GWAS analysis examined 13 ocular strains, and each of these strains had full-length *mrcA*. This analysis was expanded to include a total of 53 serovar A/B/C ocular strains that were sequenced by Hadfield and colleagues (4), and each of these additional strains also had a full-length *mrcA* (Fig. 4G). In contrast, only 20 of 180 *mrcA* sequences identified in prevalent and non-prevalent urogenital strains were full-length, with the majority having the alternate, shorter allele. Strain D/UW-3, which was the first *C. trachomatis* sequenced and annotated, is among the limited number of urogenital strains with full-length *mrcA* (Fig. 4A; Fig. S2).

Our GWAS analysis included 6 LGV strains, and each of these had a full-length *mrcA* encoding a highly similar protein (Fig. 4A; Fig. S2). The homopolymeric repeat within *mrcA* of LGV strains was interrupted by a single central G within the seven nucleotide poly-A stretch (i.e., AAAGAAA), leading to a full-length sequence containing a single amino acid change (K77E) reflective of the DNA sequence change (Fig. S2). A search of the global sequence databases led to the examination of *mrcA* in a total of 23 LGV genomes. Each of the *mrcA* sequences from LGV strains contained the AAAGAAA variant sequence (Fig. 4G).

SNPs have been identified in two genes encoding Inc proteins, leading to their inactivation (15–18). This includes *garD*, which is preferentially inactivated during *in vitro* culture of the pathogen. We examined the possibility that the altered *mrcA* sequence was similarly a product of *in vitro* growth. If this was true, only full-length *mrcA* sequences should be found in DNA amplified from *C. trachomatis*-positiv*e* specimens collected in clinics. To address this, remnant *C. trachomatis*-positive diagnostic specimens were collected and subjected to PCR-based sequencing of *mrcA*. Ten independent clinical specimens were examined, and eight of these had the truncated (6A) genotype (Fig. 4F). These data were consistent with those observed in our cultured chlamydial strains and demonstrated that the truncated *mrcA* allele was not an artifact of *in vitro* culture.

**Fig 5:**
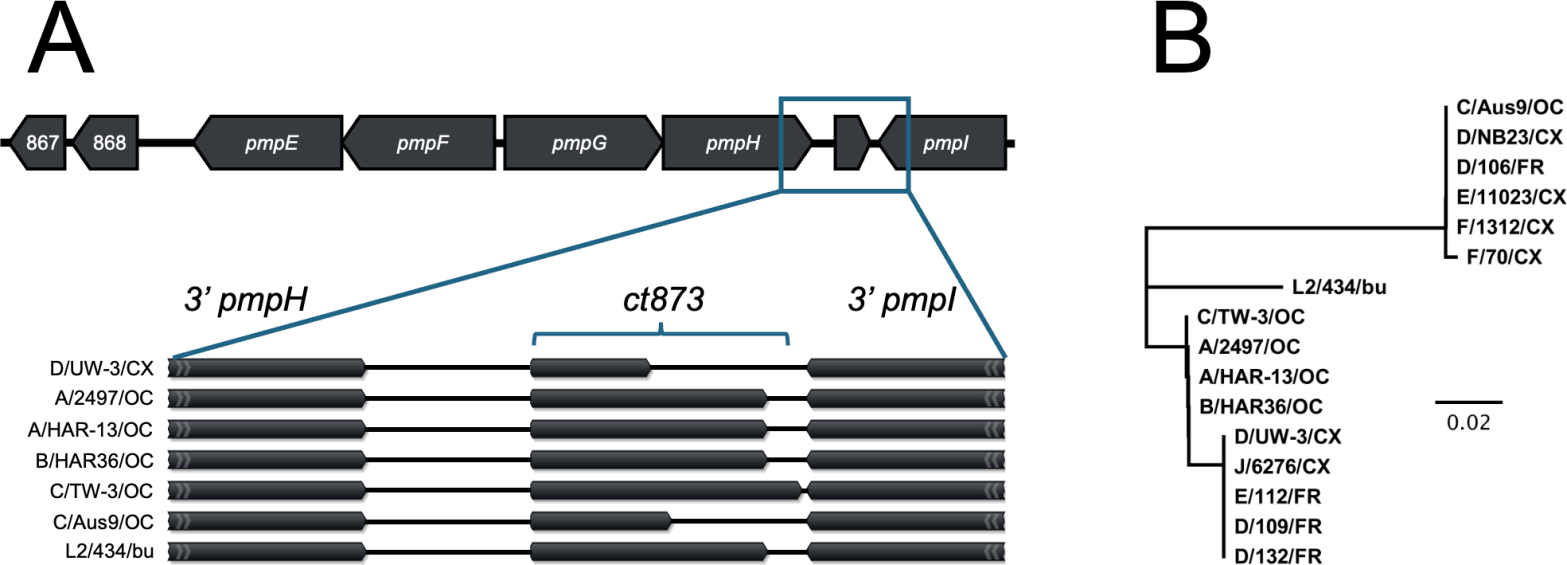
Genomic location and variability in and around *ct873*. Panel **A**: ORF map showing *ct873* between *pmpH* and *pmpI*. The expanded sequence shows that variant *ct873* ORFs are encoded by different strains. Panel **B**: Neighbor-joining-based tree showing that, with the exception of C/Aus9/OC, the ocular strains are highly conserved in this region.

Individual SNPs with high predictive value were also identified in and around *ct873*, a hypothetical gene that is present between *pmpH* (*ct872*) and *pmpI* (*ct874*). SNPs individually predictive of ocular tropism are found both within *ct873* and in the intergenic regions on both sides of the coding sequence, and some of these indels extend the length of the gene (Fig. 5). *P*-values for the mutations in and around *ct873* are very similar to those in *pmpH* and are consistent in each of the GWAS comparisons involving the ocular site of infection (Table S2, the values are highlighted in blue and gold). Additionally, GWAS values for SNPs in the very 3′ end of *pmpI* are also highly predictive of ocular tropism, but no other SNP throughout the rest of *pmpI* is tropism-predictive. The data suggest that the predictive SNPs centered on *ct873* are associated with HGT-based events leading to the acquisition of *pmpEFGH* by ocular strains and that this HGT event extends into the very 3′ end of *pmpI*.

### Identification of a genomic clade with a shared PZ genotype

The overall genome tree shown in Fig. 1A demonstrates that most lineages are defined by groups of strains expressing a single *ompA*-based serovar or genovar. The strictest example of this is in strains of serovars E or F, which are almost completely contained in single branches. The most significant inconsistency in this model is found in a major branch of the tree within the non-prevalent urogenital group. This branch, which contains strains isolated from each examined tissue source, carries isolates with at least 10 different *ompA* types and subtypes in a single genomic background. We term this branch the variable OmpA (VO) clade (Fig. 1B). Tree-building was used to examine regions of the chromosome that are shared within strains contained in the clade. A first region that was examined was the PZ, as this is a region of high variability across different chlamydial strains and species. These analyses showed that the majority of strains in the VO clade were also tightly branched in a PZ tree (Fig. 1C; Fig. S3). All but two of the VO clade strains (K/61/MU and H/SQ20/CX) are found on this branch of a PZ-focused tree.

**Fig 6:**
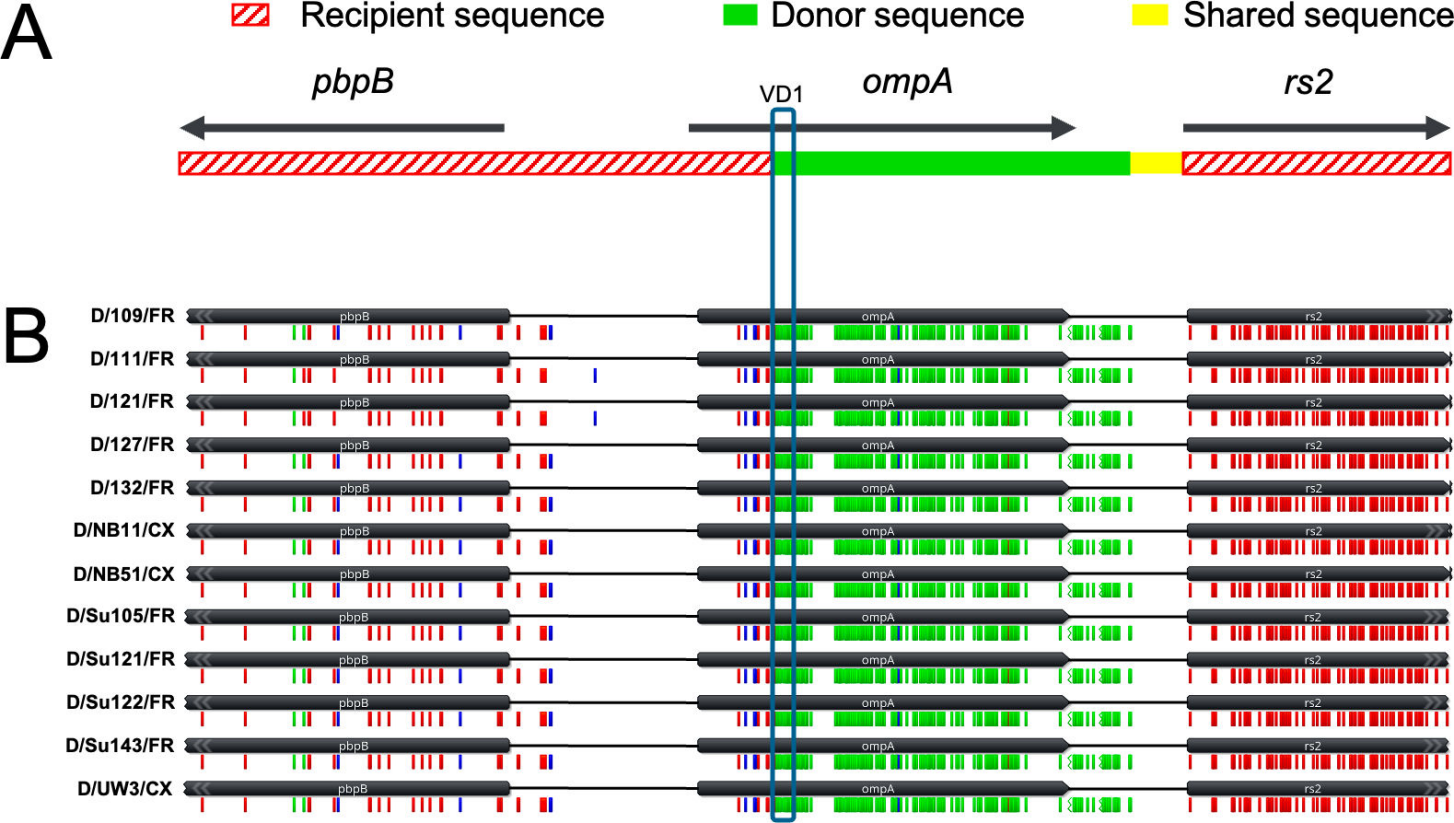
Predicted *ompA* integration positions for all serovar D VO clade strains. The analysis was conducted using strains D/NB23/CX and G/112/CX as comparators. Panel **A**: Summary of recombination positions for each integration event, with red stripes showing the recipient sequences and green showing the donor sequences. The ORFs for each gene in the region are indicated with arrows, and *ompA* VD1 is boxed. The yellow section indicates a fully shared sequence that spans the possible right end recombination site. Panel **B**: SNP-source data for each serovar D VO clade strain. Each query strain is indicated at the left. As in panel A, variant bases sourced from the recipient strain are indicated in red, while variant bases from the donor strain are indicated in green. Blue ticks indicate query strain bases that are not the same as either comparator strains.

A SNP-comparison approach was used to examine the integration endpoints for *ompA* in a set of VO clade strains. In these analyses, clade strain sequences within and around *ompA* were evaluated for SNPs that were more similar to same-serovar, non-VO clade strains, or a serotypically different strain within the clade. These analyses were most definitive when the background chromosome from likely *ompA*-donor strains was distant from strains in the VO clade, which, in these analyses, led to strains carrying B or D *ompA*. For example, strain D/UW-3/CX was used as a query strain against D/NB23/CX and G/112/MU. For VO clade strains of serovar D, the donor strain contributed sequence including all of *ompA* variable domain 1 (19) through the conserved intergenic region downstream of the *ompA* coding sequence (Fig. 6), and includes each of the four *ompA* variable domains. A similar pattern was observed for all VO clade strains that carry serovar B *ompA* (Fig. S4). The region of recombination identified between *ompA* and *rs2* has been previously reported by Gomes et al. to be a recombination hotspot (20).

**Fig 7:**
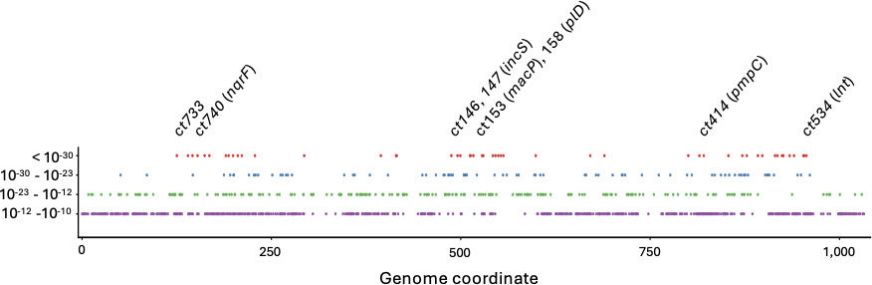
Genome coordinates of SNPs that are differently predictive of association with different genomic clades. The horizontal axis shows genome position with the scale bar indicated in kilobase pairs. The vertical axis shows the *P*-value range for each SNP along the chromosome, as shown in Table S4. The most significant *P*-values (red, top line) are predictive of members in the VO clade, while significant values between 10^−10^ and 10^−12^ (purple, bottom line) strongly correlate with the prevalent and non-prevalent urogenital clades of the tree. Coding sequences with the highest number of SNPs predictive of presence in the VO clade are indicated above the graph.

A GWAS-based computational approach was then used to identify specific loci that drive the organization of this branch of the species tree. For this analysis, genomes from strains within the VO clade were compared to genomes from all other urogenital isolates, and the significance of all identified variable sequences was determined (Table S4). Highly predictive individual SNPs are scattered across the chromosome, but genes within or at the left margin of the PZ were most predictive of the VO clade (Fig. 7). Regions with the highest predictive capability include *ct153*, encoding the *Chlamydia* perforin homolog MacP, *ct158*, which encodes a phospholipase D*,* and *ct146-147*, which encode a predicted DNA ligase and an inclusion membrane protein (IncS (21, 22)), respectively.

The coding sequence of *ct158* is another example of a single indel that is associated with predicted functional or structural differences in a protein. Sequence comparisons of different alleles of *ct158* identify an indel at position 474 that alters its reading frame. In VO clade strains, this reading frame is intact and encodes two HDK motifs {HXK(X)_4_D(X)_6_GSxN} (Fig. S5), both of which, in other systems, are required for phospholipase activity (23). Enzymes having only a single HDK motif lack lipase activity but can have endonuclease activity (24). All *C. trachomatis* strains with an insertion at position 474 encode only a single HDK motif. The indel in *ct158* is highly predictive of localization to the VO clade, with 45 of 46 strains carrying a gene containing both HDK motifs.

## DISCUSSION

The present study was initiated to expand our previous GWAS-based analysis, which showed tissue-tropic sequence differences between *C. trachomatis* cultured from the male rectum vs. those collected from the female lower genital tract (10). Sequence polymorphisms within a set of three loci*—pmpEFGH*, *ct144,* and *ct050*—were associated with tropism to these tissues. This comparison left open the possibility that the observed male rectal tropism was truly focused on that tissue or perhaps that similar strains would also be enriched in the female rectum and/or the male urethra. The GWAS experiments described in this work examined 200 genomes from strains collected from each of five tissue sites. The data demonstrated that strains isolated from the male rectum had a unique genomic signature relative to strains isolated from either female anatomic site or from the male urethra. Additionally, comparisons of male rectal strains vs. either female rectal, cervical, or male urethral strains were highly similar, demonstrating that *pmpE*, *pmpF*, and *ct144* were indeed tropism-predicting for comparisons of strains isolated from these tissues. Therefore, these data support the hypothesis that *C. trachomatis* strains isolated from the male rectum are unique relative to those cultured from other tissues involved in sexually transmitted infections. Work by other individuals has demonstrated that each of these proteins—PmpE, Pls1, and CT144—is localized to the lumen of the inclusion within infected cells (25–27). This leads to the surprising hypothesis, wherein tissue tropism may be determined by unknown intra-inclusion factors or processes that are distinctly different between tissue sites.

The comparison between male urethral and female rectal strains represents a significant outlier in both the GWAS and the subsequent PCA described in this work. In the GWAS, the set of genes identified as significant in this comparison are primarily strains that separate the prevalent and non-prevalent urogenital groups and are unique with respect to any other GWAS comparison in this study (Fig. 2). These differences are reflected in the multivariate analysis, in which the female rectal vs. male urethral comparison is significantly different than any other comparisons (Fig. 3). We have no behavior data for our deidentified samples. The male urethra is a tissue that is not uniquely involved in any single epidemiologic or behavioral pattern associated with *C. trachomatis* infection, and the microbiome of this tissue is modest and influenced by sexual behavior (28). The lack of behavior or sexual preference information for our male urethral samples may play a role in the unusual GWAS data associated with male urethral vs. female rectal genome sequence. Future studies will address male urethral strains in individuals for whom sexual behavior patterns are known.

Our GWAS analyses also included *C. trachomatis* collected from the ocular site of infection. Comparison of ocular isolates with all others led to more complicated profiles than those observed in the STI comparisons, but each comparison in the group is very consistent (Fig. 2G through J). The GWAS profiles involving ocular strains are supported by PCA, which shows that all GWAS comparisons involving ocular strains are statistically different than all comparisons not involving ocular strains. While our GWAS comparisons include a very limited number of ocular strains, our data are very similar to the work of others (11).

Most of the tropism-predicting sequence in the species can be quantified using a gene-centered approach. However, there are examples of single SNPs that are highly predictive, particularly regarding strains infecting the eye. Single-base changes in *mrcA* and in the region between *pmpH* and *pmpI*, most often manifested as an indel, led to clear differences in GWAS-predicted tissue tropism to the ocular site of infection. In each of the two most statistically significant of these, an indel changes the reading frame but leads to a protein that is not immediately truncated. An examination of the literature demonstrates that there are at least two examples of polymorphisms in chlamydial *inc* genes that lead to differential expression phenotypes in strains. The first of these is *incA*, in which a variety of inactivating mutations can be found in urogenital strains, leading to the absence of IncA on the inclusion membrane and an associated inclusion fusogenicity defect (15, 29). The second sequence is *ct135* (*garD*), in which inactivating mutations accumulate in lab-grown strains, leading to cell autonomous sensitivity of these strains when grown in the presence of interferon gamma (16–18). We hypothesize that these changes might lead to protein species with different functions in the different tissues, as opposed to the indel leading to full inactivation of the out-of-frame allele. For example, the two proteins generated by the two *mrcA* alleles are each correctly localized to the inclusion membrane (Fig. 4E and F), although their predicted cytosol-exposed functional domains are very different in sequence (Fig. S2) and predicted structure (Fig. 4B).

The VO clade is a group of strains with a shared genomic backbone and a wide variety of *ompA* genotypes. This is in contrast to many of the terminal branches of the tree, in which strains with a shared genomic background most often carry a single *ompA* type (Fig. 1; Fig. S1). With the exception of serovars E and F, there are examples of each urogenital *ompA* type in the VO clade, including the less common *ompA* types, such as Da, H, I, and K (Fig. 1B). The VO clade also includes isolates expressing serovar B *ompA* sourced from four different body sites, including the eye. VO clade strains expressing the extremely rare serovar I *ompA* allele were also isolated from four anatomical sites of infection (cervix, male urethral, female urethral, and female rectal).

GWAS-based analysis demonstrated that sequences most highly predictive of the VO clade reside within or just to the left of the *C. trachomatis* PZ (Fig. 7), and there are short, predictive SNPs in other genes such as *pmpB* and *pmpC*. A single indel in *ct158* is highly predictive of presence in the VO clade, with 45 of 46 strains in the clade having a shared sequence at this position. This sequence is predicted to encode an intact, functional phospholipase D, while a majority of non-clade strains (124/154) do not encode a protein with predicted phospholipase activity.

It is challenging to speculate why the VO clade might attract such a wide variety of *ompA* types. We hypothesize that this clade has a selective advantage in contemporary *C. trachomatis* natural history and that its success has led to contact with other strains in coinfections and HGT-based acquisition of different *ompA* sequences. Although the nature of a protective anti-chlamydial immune response is very complicated (30), it is possible that the acquisition of various *ompA* types in a single genomic background might lead to the avoidance of immune responses in individuals with repeat infections by VO clade strains. There is historical evidence of serovar-specific immunity in humans (31), and contemporary work with macaques shows that very minor sequence changes in OmpA can lead to the lack of neutralizing antibody responses (32). This speculation is preliminary, however, and the driving force leading to such a broad collection of *ompA* types in an otherwise tight genetic linkage remains an open question.

Examination of the phylogenetic tree demonstrates that the VO clade is closest to a cluster of serovar G strains isolated from the cervix, female rectum, and male urethra (Fig. 1; Fig. S1). This linkage supports a hypothesis that HGT-based genomic changes led to the initial branching of the clade away from the urogenital serovar G strains. Each of these groups is a member of the broader non-prevalent urogenital clade, and each shares selected loci acquired via HGT, such as *ct144* and *pmpC*. However, these groups do not share overall PZ similarity nor have the G strains acquired clade-specific alleles for *ct146*, *ct147* (*incS*), and *ct153*. In contrast, the male rectal Ga/J branch shares similarity with the VO clade strains in the PZ and, especially, in genes *ct157-159*. (Fig. 1C; Fig. 7). We hypothesize that the clade progenitor strain/strains acquired unique PZ-associated alleles, perhaps from a serovar Ga or J strain that, in total, led to a competitive advantage in an undefined STI niche. A second level of selection then led to the accumulation of different *ompA* types, possibly for reasons associated with immunity in repeat infections.

## MATERIALS AND METHODS

### Strain collection and DNA preparation

The majority of the specimens and the resulting chlamydial cultures used in this study were collected from individuals attending Seattle area sexual health clinics between 1982 and 2010 (10). Individual strains were chosen based on a confirmed anatomical site of specimen collection, followed by a consideration of overall serovar representation. Each strain is labeled with a coding system as follows: Serovar or genovar/sample name or number/tissue collection site. The tissue coding throughout is as follows: female genital tract isolates, CX; female rectal isolates, FR; male urethral isolates, MU; male rectal isolates, MR; and strains collected from the conjunctivae, OC. Thus, strain D/UW-3/CX is a serovar D strain originally labeled UW-3 and was cultured from the cervix. Our previously published methods were used for initial culture, expansion, and purification of clinical strains (33). Genomic DNA was purified from McCoy-cell-propagated EBs, basically as described previously (10). Briefly, purified EBs were sonicated and then treated with RQ1 DNAse (Promega, Madison, WI) to reduce host cell DNA. Chlamydial elementary bodies were then lysed with detergent and dithiothreitol, and DNA was purified using the Qiagen Blood and Tissue Kit (Qiagen, Germantown, MD).

### DNA sequencing, genome assembly, and phylogeny reconstruction

Genome sequencing was conducted on an Illumina platform at the Oregon State University Center for Quantitative Life Sciences. All genomes were assembled in Geneious using its mapper script (34). The process was initiated by generating reference-guided assemblies against serovar-specific reference genomes. After each assembly pass, quality-filtered, trimmed reads were remapped in a single high-sensitivity iteration to evaluate and validate the results. Gaps attributable to insertions or deletions were resolved by *de novo* contig assembly in Geneious with Velvet v1.2.10 (34). Intra-isolate variation was identified using FreeBayes v1.3.2-38 (35), and genomes were polished as needed. For comparative analyses, whole genomes from 200 isolates (Table S1) were aligned in Geneious with MAFFT v7.450 (36). The *C. muridarum* strain VR-123 genome sequence was included as an outlier. Genes implicated by the GWAS were extracted from the WGS alignment within Geneious. Phylogenetic trees for both the whole-genome and gene-specific alignments were inferred using the Geneious Tree Builder with the Jukes-Cantor distance model and the neighbor-joining method.

### Genome-wide association studies

All methods are as originally described by Suchland et al. (10). Quality-assembled whole genome DNA sequences were obtained from 192 clinical samples (Table S1), each labeled by the site of isolation. Whole genome sequences were aligned against a reference genome assembly sequence (D/UW-3/CX) using MAFFT Aligner (*v7.490*) within Geneious (36). Variants were annotated using Geneious variant caller, providing a full genomic database of individual sample variants against the reference. To investigate how specific pathogen genes may influence propensity for chlamydia infection in different human host tissues, we used a GWAS approach that examined all isolates from different tissues and then quantified the number of statistically significant genetic variants on a per-gene basis (10,981 total variant positions), using methods described previously (10). This collection of mutations was then plotted on the D/UW-3/CX reference genome sequence, and variants were integrated with the alignment map of the isolates’ genomes. The significance of the collected variants in each of these genes was then corrected for the number per kilobase of gene length. The genome coordinates were then redefined in order to place the start position consistent with that of the L2/434 genome, which starts at *hemB* (CTL0001). Values representing the significant SNPs/gene/kb for each of the tissue comparisons were then plotted against the overall chromosomal sequence.

### Principal component analysis

Using a custom R script, we organized the gene-level significance profiles of the 10 tissue type comparisons as a 776 × 10 matrix and processed the matrix via PCA using the built-in R function *prcomp*. We then carried out the multivariate difference-of-means test between the two clusters of tissue type comparisons (Fig. 2) using a permutation test of Hotelling’s $T^{2}$ statistic, calculated using the ten tissue comparisons' scores for principal components 1 and 2, in R using the software package Hotelling (version 1.0-8).

### Fluorescent antibody labeling

An antiserum against *C. trachomatis* MrcA was used to demonstrate the presence and localization of MrcA within infected cells (antisera graciously provided by Dr. Ted Hackstadt, NIH). For these experiments, murine McCoy cells were cultured on 1 cm glass coverslips on 24-well-tray wells, followed by infection with different *C. trachomatis* strains. Infected cells were incubated in standard conditions (33) for 24–30 h, prior to methanol fixation and antibody labeling. Appropriate secondary antibodies were purchased from Southern Biotech (Homewood, AL), and DNA was labeled with DAPI. Labeled monolayers were viewed on a Leica DMLB under oil immersion. Images were collected using a Leica camera and its associated software package.

## SUPPLEMENTAL MATERIAL

Supplemental figures iai.00266-26-s0001.pdf Fig. S1 to S5.

Table S1 iai.00266-26-s0002.xlsx Strain data.

Table S2 iai.00266-26-s0003.xlsx GWAS data.

Table S3 iai.00266-26-s0004.xlsx ct101 SNPs.

Table S4 iai.00266-26-s0005.xlsx VO clade GWAS data.

ASM does not own the copyrights to Supplemental Material that may be linked to, or accessed through, an article. The authors have granted ASM a non-exclusive, world-wide license to publish the Supplemental Material files. Please contact the corresponding author directly for reuse.
