# Supplementary material for: Genome-wide association studies of C. trachomatis identify novel tissue tropism-predicting loci and sequences that discriminate among major genome sequence clades
Source: Infect Immun. 2026 Jul 17;94(8):e00266-26. doi: 10.1128/iai.00266-26 (PMC13459586; doi:10.1128/iai.00266-26)
Supplement: Supplementary file 1 [file iai.00266-26-s0001.pdf]

Supplemental figures for

Genome-wide association studies of *C. trachomatis* identify novel tissue tropism-predicting loci and sequences that discriminate among major genome sequence clades.

Suchland et al., 2026

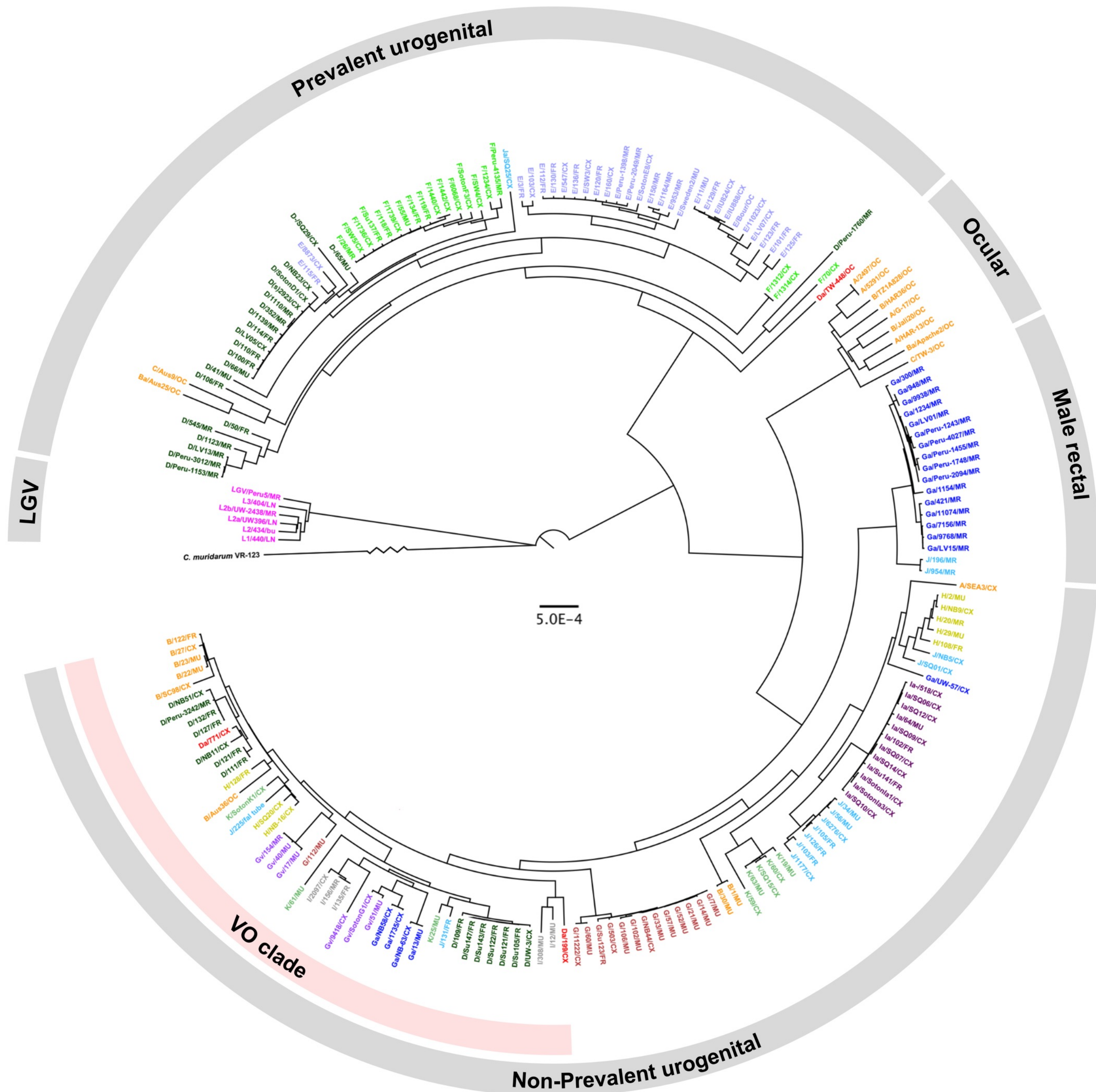

Figure S1. Neighbor-joining-based phylogenetic tree of 200 *C. trachomatis* isolates, with each strain name indicated in the tree. The *Chlamydia muridarum* VR-123 chromosome is included as an outlier. Each major clade is indicated in the gray arcs. The VO-Clade is indicated with the pink arc. Color coding of strains is as indicated in Figure 1.

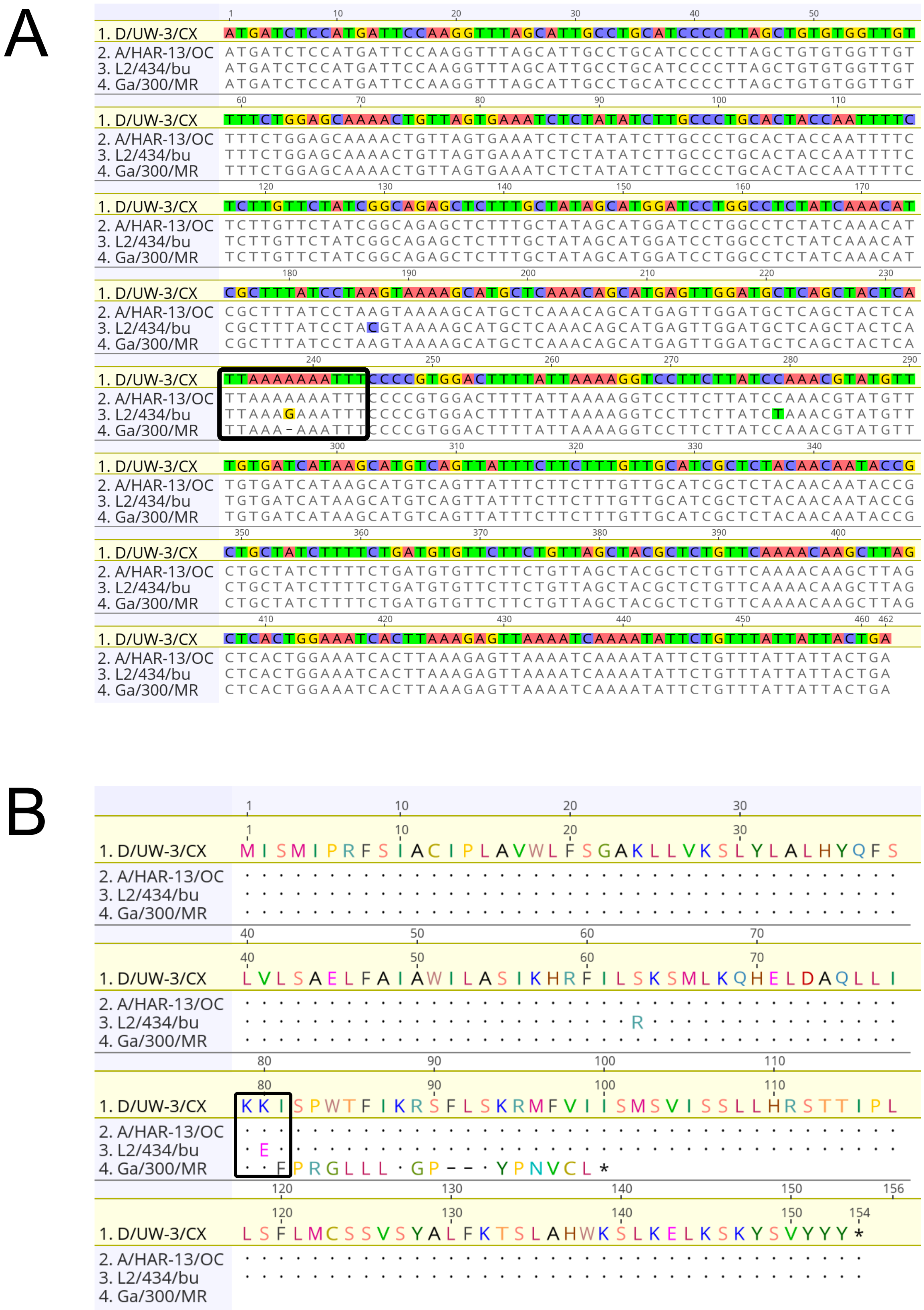

Figure S2. DNA (A) and protein (B) alignment of *mrcA* (*ct101*) and its gene product in strains that have sequence differences in the poly-adenosine repeat. The region surrounding the polymorphism is boxed in each panel.

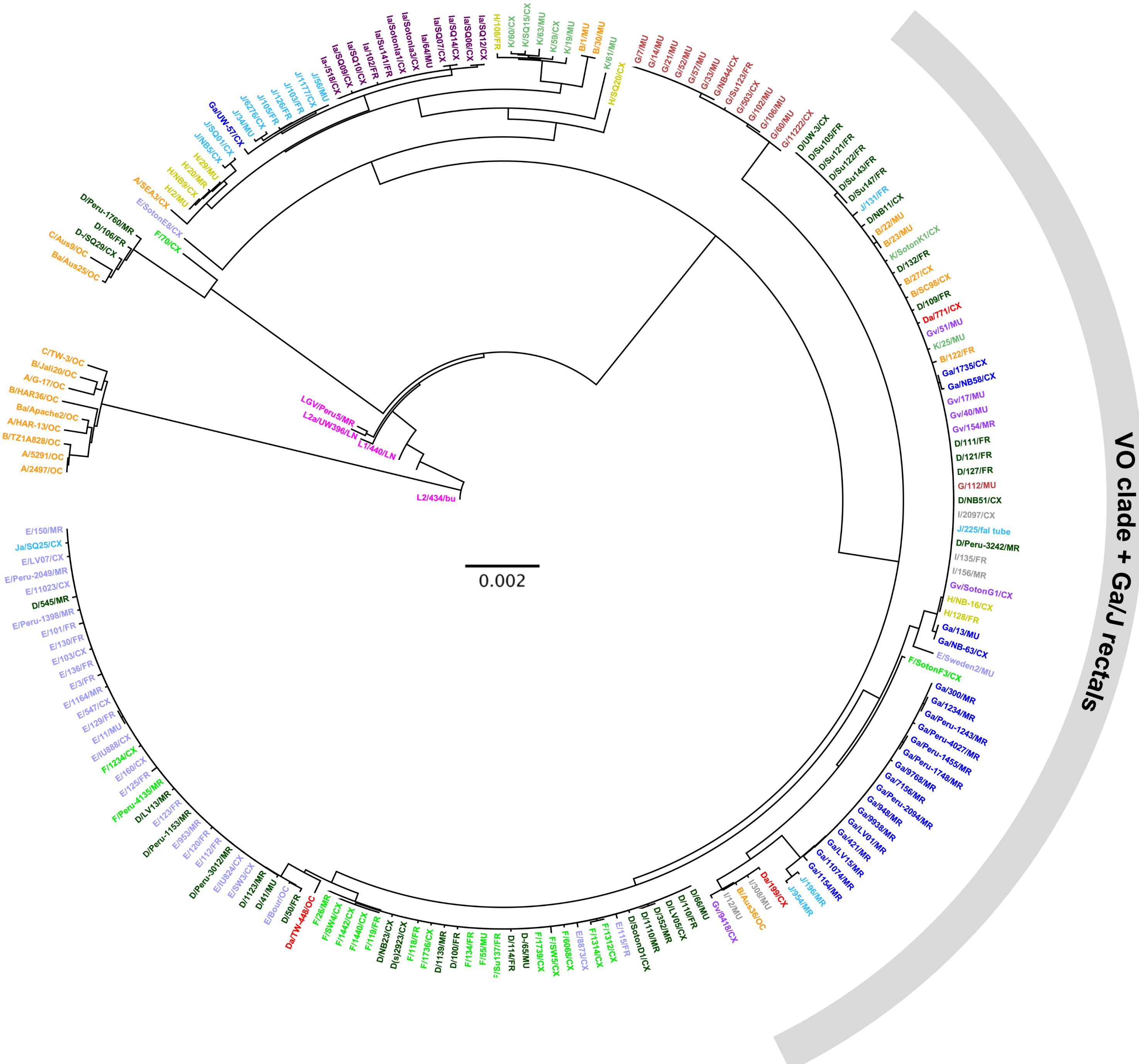

Figure S3. Neighbor-joining-based phylogenetic tree based on the *C. trachomatis* plasticity zone (*ct154-ct174*). Each strain in Figure S1 and Table S1 is included in this analysis. Color-coding is as shown in Figure 1.

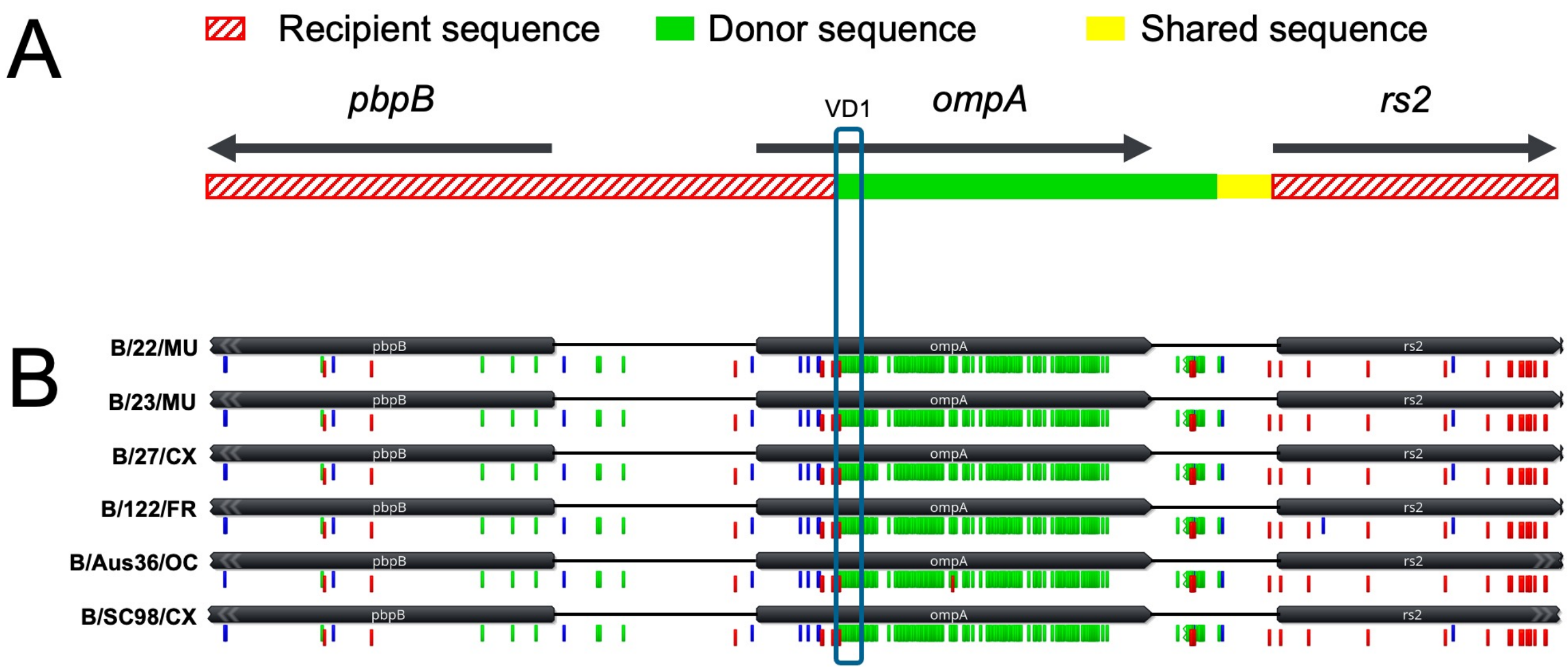

Figure S4. Predicted *ompA* integration positions for all all serovar B VO-clade strains. The analysis was conducted using strains B/HAR36/OC and G/112/CX as comparators. The vertical box indicates the predicted left margin of recombination, within *ompA* and upstream of the first variable domain, VD1. Panel A summarizes the recombination positions for each integration event, with red stripes showing the recipient sequences and green showing the donor sequences. The open reading frames for each gene in the region are indicated with arrows, and *ompA* VD1 is boxed. The yellow section indicates fully shared sequence that spans the possible right end recombination site. Panel B. SNP-source data for each serovar D VO-clade strain. Each query strain is indicated at the left. As in panel A, variant bases sourced from the recipient strain are indicated in red, while variant bases from the donor strain are indicated in green. Blue ticks indicate query strain bases that are not the same as either comparator strain.

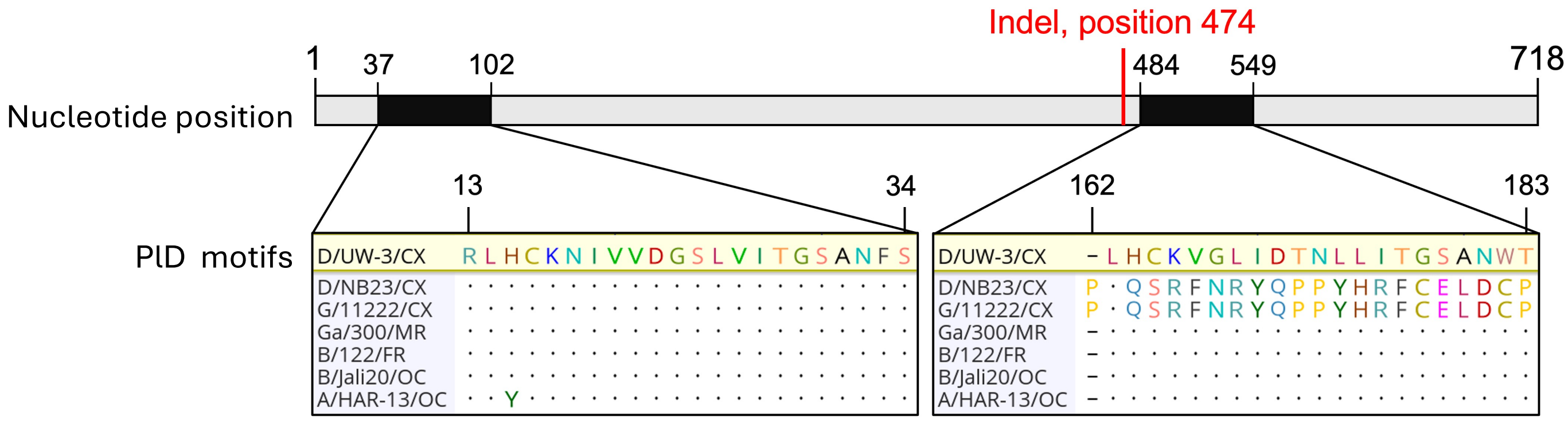

Figure S5. Cartoon of the *C. trachomatis* phospholipase D (*ct158*) full length coding sequence and the position and sequence of PlD motifs (HXK(X)$_4$D(X)$_6$GSxN). The indel that leads to a frameshift in the sequence is found at nucleotide position 474, and this leads to the elimination of the second PlD motif in most urogenital strains outside of the VO-clade.
